# Value-sensitive design and global digital health

**DOI:** 10.2471/BLT.19.237362

**Published:** 2020-07-08

**Authors:** Karin R Jongsma, Fleur Jongepier

**Affiliations:** a Julius Center, PO Box 85500, 3508 GA Utrecht, University Medical Center Utrecht, Utrecht University, Netherlands.; b Faculty of Philosophy, Theology, and Religious Studies, Radboud University, Nijmegen, Netherlands.

In the field of digital ethics, value-sensitive design^1^ is one of the most influential normative methodologies. The basic aim of value-sensitive design is to integrate human values in technologies from the very start of the design process.^1^ While value-sensitive design is widely discussed and applied to various technologies in the western European context, in low- and middle-income countries this approach is only rarely applied. This neglect is surprising, as the hopes for digital health are especially high in the context of global health.^2^^,^^3^

Local values, habits and practices that are shaped by local circumstances, including socioeconomic contexts, influence which digital technologies get adopted and how they are used. For instance, in Bangladesh, mobile phones are often shared with family members, partners and children due to economic conditions, but also because sharing is a deeply embedded social and cultural practice.^4^ A digital health application for a community of users in Bangladesh should thus be aligned with these local values, including notions of privacy, rather than designing privacy features based on the western assumption that a mobile phone only has one user.^1^ Value-sensitive design may be helpful to integrate such local practices and values in developing digital technologies in low- and middle-income countries.^5^ Our aim here is to explain the approach of value-sensitive design, illustrating its strengths with an example, and to flag some important limitations and challenges of this approach.

## Value-sensitive design

Value-sensitive design emerged in the 1990s as a reaction to the principal focus of engineers on functionality and efficiency, with researchers suggesting that other values should be incorporated to technology design. This approach advocates for frontloading ethics,^6^^,^^7^ that is, addressing the ethical implications before the technology has been fully developed. Given that technology is never value-neutral, these researchers argue that it is better to explicitly integrate values into the design process, whereby a value is defined as what “a person or group of people consider important in life.”^1^ Privacy, well-being, ownership and property, freedom from bias, autonomy and consent, are all mentioned as values, these are also prominently discussed as leading values in the digital health context.^8^

Value-sensitive design employs an iterative approach that integrates conceptual, empirical and technical investigations as repeating parts of the process.^1^ The first, conceptual phase encompasses identifying and defining the relevant values involved, as well as mapping direct and indirect stakeholders. The second, empirical stage of value-sensitive design involves providing information about the human context in which the device will be used by drawing on quantitative and qualitative methods of the social sciences (for example, conducting interviews and surveys). Finally, technical investigations focus on how existing technological properties and underlying mechanisms of the technology support or hinder human values, and on translating stakeholder values into the technical features of the device.

Value-sensitive design has rarely been applied in low- and middle-income countries, but one example can be found in a programme that was aimed at the delivery of vaccines in rural areas and was facing supply problems.^5^ The programme involved several stakeholders, involved directly in these vaccine delivery programmes, as well as indirectly, such as local nongovernmental organizations. Based on the stakeholder analysis, researchers identified several important values, including accountability, respect, autonomy, trust, sharing and access. The researchers subsequently chose to focus on the values of respect and accountability, by suggesting that these values affected all involved stakeholders and were considered directly relevant for the improvement of the programme. The researchers then proceeded to the technical investigations of the value-sensitive-design approach, rather than moving to the empirical investigations, as is more common for a value-sensitive design approach. The technical investigations involved identifying the best way for health-care clinics to report that they were out of supply, including adjustments to hardware flexibility and the level of data aggregation. In a mock-up design of the new vaccine delivery system, different ways of redesigning this system and options for communication were added, including language choices. These technical adjustments resolved one of the existing system’s problems, where people interacting with the system did not speak the programmed language. Several questions to identify the status and source of lack of supply were added, including the option to answer “I don’t know.” However, local nongovernmental organizations pointed out that answering one of the required questions of the communication system with “I don’t know” had a completely different, and very negative, connotation in the local language. The researchers reported that the empirical investigations were still ongoing at the time the paper was published and were aiming at establishing mutual respect and trust with the stakeholders working with the systems. The input of stakeholders was foreseen to inform the technological design and to resolve tension between important values and the future potential of the system.

## Strengths

Assessing how technologies align with norms and values is crucial to value-sensitive design, particularly when applied to a global context. Local practices may exemplify different understandings of key values such as autonomy, respect and privacy. Depending on the circumstances, however, different aspects of a technology (including those related to programmed language and functionalities) might have to be adjusted to enable its adaptation to a specific context. The example about the different connotations of certain expressions in different languages, for instance, indicates that language and phrasing require attention to reinforce values of respect and accountability. In general, the empirical reality, that is the actual context in which the technology will be used and how it will be used, can have implications for how certain concepts are understood and valued (such as different meanings of privacy in different contexts). Technological design needs to respond to and align with local norms and values to resonate with the lived experiences and local conceptions of users, and additionally, may contribute to acceptance of the technology. The technical stage of value-sensitive design includes exploring whether the design’s goals can be fulfilled, for example whether the required bandwidth or phone connection that the digital health application requires is available in the first place.^8^

## Limits and challenges

While value-sensitive design has the potential to provide normative guidance and improve the development of digital health technologies in a global health context, the approach also presents challenges and potential shortcomings. Most importantly, not all values are morally relevant values.^6^^,^^9^ For instance, some authors mention morning tea and a walk in the woods as values. We suggest the first stage of value-sensitive design could be improved if it explicitly involved an investigation of relevant moral values. What defines moral values or norms is that they override other norms or values. Non-moral values can be part of a value-sensitive design-based analysis, but only as factors to be mentioned in a trade-off (for instance, costs and user satisfaction). In the vaccine programme example, one might wonder whether the values of sharing and access are moral values, and if so, why exactly.

Second, although the founders of value-sensitive-design emphasize the iterative nature of their approach,^1^^,^^6^^,^^9^ the strict division of conceptual, empirical and technological investigations risks undermining this iterative character. This may also give the impression that identifying and defining key moral values can be done pre-empirically, that is, without exploring and understanding the local context, needs and practices beforehand. In the example provided, the choice for focusing on the central values of respect and accountability was made by the researchers, rather than by the stakeholders. As we outlined above, one might, as part of a conceptual investigation, include empirical investigations on how specific stakeholders understand certain concepts and what values are most important to them, or on how using a certain technology has changed their conception of it.

Third, and particularly relevant in the global context, stakeholder analysis is part of the conceptual phase of value-sensitive design.^1^ However, when this analysis is not supplemented with solid empirical identification of stakeholders, the technological design could risk being insensitive to local hierarchies, failing to anticipate how people will be affected by the technology and to identify some essential, but less visible stakeholders. Such negative outcomes could cause harm, by reaffirming problematic (for instance, potentially oppressive) hierarchies and the marginalization of those most in need.

Finally, a crucial action in this approach and its applications is missing, namely, to justify the relevant moral values, and not only identify and define them. This justification also involves explaining which moral considerations or theories should form the basis for trade-offs between conflicting moral values (for instance between health, privacy or accountability).^10^^,^^11^ Part of the justificatory aspect of value-sensitive design is also the question of what weight to assign to local values and practices, and how to factor in local values when they threaten other values, such as well-being or autonomy. Without adequate justification, any value-sensitive analysis would at best only reflect the potentially arbitrary values of stakeholders (or researchers). At worst, the analysis would only rephrase the original situation: many values are involved, they are hard to define, and some are incompatible with others. Constructing a value-sensitive design-based analysis is challenging conceptually, empirically and technically, but if conducted properly, may provide much-needed empirically-informed normative guidance. 

## References

[1] Friedman B, Kahn PH Jr, Borning A. Value sensitive design and information systems. In: Zhang P, Galletta D, editors. Human-computer interaction in management information systems: foundations. Armonk: M.E. Sharpe; 2006 pp. 348–72.

[2] Recommendations on digital interventions for health system strengthening. Geneva: World Health Organization; 2019. Available from: https://www.who.int/reproductivehealth/publications/digital-interventions-health-system-strengthening/en/ [cited 2020 Jun 26]. 31162915

[3] Asfaw S, Morankar S, Abera M, Mamo A, Abebe L, Bergen N, et al. Talking health: trusted health messengers and effective ways of delivering health messages for rural mothers in Southwest Ethiopia. Arch Public Health. 2019 2 21;77(1):8. 10.1186/s13690-019-0334-4 30828451 PMC6383212

[4] Ahmed SI, Haque R, Chen J, Dell N. Digital privacy challenges with shared mobile phone use in Bangladesh. Proc ACM Hum Comput Interact. 2017 11;1(CSCW):17 10.1145/3134652

[5] Walton R, Derenzi B. Value-sensitive design and health care in Africa professional communication. IEEE Trans Prof Commun. 2009;52(4):346–58. 10.1109/TPC.2009.2034075

[6] Manders-Huits N. What values in design? The challenge of incorporating moral values into design. Sci Eng Ethics. 2011 6;17(2):271–87. 10.1007/s11948-010-9198-2 20224927 PMC3124645

[7] Hoven J. Design for values and values for design. Information Age. 2005;4:4–7.

[8] Lucivero F, Jongsma KR. A mobile revolution for healthcare? Setting the agenda for bioethics. J Med Ethics. 2018 10;44(10):685–9. 10.1136/medethics-2017-104741 29907579 PMC6173811

[9] van de Poel I. Design for value change. Ethics Inf Technol. 2018 10.1007/s10676-018-9461-9

[10] Arras J. Theory and bioethics. The Stanford Encyclopedia of Philosophy. Standford: Metaphysics Research Lab, Stanford University; 2016 Available from: https://plato.stanford.edu/entries/theory-bioethics/ [cited 2020 Jun 17].

[11] Jacobs N, Huldtgren A. Why value sensitive design needs ethical commitments. Ethics Inf Technol. 2018 7;1–4: 10.1007/s10676-018-9467-3

